# A pilot study on early microgliogenesis following unilateral vestibular neurectomy: A key player in vestibular compensation?

**DOI:** 10.1371/journal.pone.0339767

**Published:** 2026-01-07

**Authors:** Jessica Trico, Emna Marouane, Alain Tonetto, Isabelle Watabe, Agnes Lapotre, Brahim Tighilet

**Affiliations:** 1 Aix Marseille Univ, CNRS, CRPN (Centre de Recherche en Psychologie et Neurosciences - UMR 7077), Marseille, France; 2 Normandie Université, UNICAEN, INSERM, CYCERON, CHU Caen, COMETE UMR, Caen, France; 3 Aix Marseille Univ, CNRS, Centrale Med, FSCM (UAR1739), Plateforme de Recherche Analytique Technologique et Imagerie (PRATIM), Marseille, France; Universiti Malaya, MALAYSIA

## Abstract

Following unilateral vestibular damage, several behavioral deficits arise, referred to as vestibular syndrome. At the central level the vestibular syndrome is associated with an imbalance in neuronal activity between the two vestibular nuclei (VN). Its recovery is correlated with a rebalancing of the electrophysiological activity between both VN, known as vestibular compensation. Key plasticity mechanisms within the VN involved in this mechanism include, among others, neurogliogenesis, modulation of neuronal excitability and increased histamine release. In this study, we aimed to characterize the acute glial cell differentiation lineage in response to unilateral vestibular neurectomy (UVN) in the deafferented VN. We further assessed whether this response is influenced by the histaminergic system. To achieve this, betahistine dihydrochloride (BD), was used to stimulate histamine synthesis and release in the VN. After UVN, 2 animal groups were treated orally during 3 days with either BD treatment (UVN BD group, 50 mg/kg/day) or placebo (UVN placebo group). We present preliminary evidence of acute and abundant microgliogenesis restricted to the deafferented VN in both groups. This phenomenon does not appear to be mediated by BD treatment but may reflect an intrinsic biological adaptative mechanism. Further investigations, including Sholl analysis, would be essential to characterize microglia, which may represent a key player in vestibular compensation.

## Introduction

The loss of input from one vestibular organ induces an imbalance between the two vestibular nuclei (VN), producing characteristic postural and oculomotor symptoms. Functional recovery, known as vestibular compensation, relies on various neuroplasticity mechanisms within the deafferented VN over time to restore electrophysiological symmetry between the lesioned and contralateral sides [1–6].

Glial activation and cell proliferation are both component of this remarkable plasticity mechanisms [7–11]. Previous work from our team demonstrated an acute and long term glial activation within the deafferented VN [9,10,12,13]. This glial activation has also been reported in other vestibular lesion models [7,8,14,15] supporting its involvement in vestibular compensation. However, cell proliferation have been reported only in unilateral vestibular neurectomy (UVN) model [9,11,16,17]. Interestingly, both pharmacological compounds and rehabilitation protocols used to improve balance function have been demonstrated to favor microgliogenesis at the expense of neurogenesis [10,12,13].

In our previous investigation, we demonstrated that a pro-histaminergic drug (betahistine dihydrochloride, BD), modulated central vestibular plasticity and promoted the restoration of posturo-locomotor functions [13]. In this previous study, cell lineage analysis of proliferating cells was conducted only one-month post-UVN, revealing a predominance of microglial cells with BD treatment. However, this plasticity mechanism remains to be elucidated during the acute phase of the vestibular syndrome as this period is considered a likely critical period in which important neuroplasticity mechanisms take place to promote vestibular functional recovery [18,19].

The present work is a follow-up of the previous study [13] and provides preliminary evidence on proliferating glial cells during the acute stage of the vestibular syndrome in UVN rats either treated or untreated with BD. Using high resolution confocal microscopy, we examined BrdU-labeled proliferating cells co-expressing glial markers (GFAP, Olig2, IBA1) within the VN.

## Materials and methods

### Animals and ethics statement

Ten (10) female Long Evans rats (10–12 weeks old, 250–300 g) from our in-house breeding, originally derived from Charles River (St Germain sur l’Arbresle, France) were included in this study. All experimental procedures were conducted under veterinary and National Ethical Committee supervision. The experimental protocol was evaluated by the Neurosciences Ethics Committee No. 71 of the French National Committee for Animal Experimentation and received official written approval from the French Agriculture Ministry (Project Authorization No. 24896–2020031314304407). All personnel participating in this project possessed the mandatory French certification for animal experimentation and were trained in animal welfare and care. All surgery was performed under isoflurane anesthesia and efforts were made to reduce both the number of animals used and their suffering throughout the experiment. Rats were housed at the Fédération 3C (Centre Saint-Charles, Aix-Marseille University) animal facility, under a 12 h light/dark cycle, with ad libitum access to food and water. Two animals were excluded from the study (see Surgery section). Animals (n = 8) were assigned to two groups: UVN placebo group (n = 3) and UVN BD group (n = 5). Detailed descriptions of the surgical procedures and experimental protocols are provided below.

### Study design

All 10–12 weeks female rats were manipulated for 7 days before UVN and assigned to two groups (UVN placebo n = 3 and UVN BD n = 5). UVN BD group (n = 5) received 50 mg/kg/day by oral gavage for three consecutive days, while UVN placebo group (n = 3) underwent gavage with an equivalent volume of 0.9% sodium chloride solution. To evaluate cell proliferation, all rats received an intraperitoneal BrdU injection (bromodeoxyuridine, 200 mg/kg) 3 days after the UVN and were sacrificed 2 hours later, as described in our previous report [13]. The 2-hour BrdU incorporation represents a short observation time, yet it is sufficient to label a substantial number of proliferating cells, as previously observed in other studies using the UVN model [11–13,16,17,20]. Behavioral assessments for these animals were performed in a separate study [13] using the same groups and are not included here.

### Surgery

The UVN was performed on 10 rats following the surgical procedure previously described in the literature [21]. Buprenorphine (Buprecare®; 0.05 mg/kg) was administrated subcutaneously 30 minutes prior to surgery. Rats were deeply anesthetized with isoflurane (4% for induction, 3% for maintenance). To compensate for potential fluid losses during surgery, Ringer Lactate solution (Virbac; 10mL/kg) was injected subcutaneously before awakening. Successful surgery was confirmed by awakening with the onset of characteristic vestibular symptoms. Animals without symptoms at awakening were excluded from the study (n = 2). During the 3 days following the lesion, animals were monitored at least twice daily using a standardized animal-welfare scoring system. Humane endpoints—based on grooming, natural behavior, hydration status, clinical signs such as breathing/ventilation, response to handling, and anorexia (> 20% loss of pre-operative body weight)—triggered immediate euthanasia. No animal in this study reached these endpoints, and all animals were perfused at the end of the experiment for immunohistochemical analysis.

### Tissue preparation

Anesthesia was induced by intraperitoneal injection of ketamine (Imalgene 1000®; 100 mg/kg) and medetomidine (Domitor®; 0.5 mg/kg). Rats were then perfused intracardially with 400 mL of isotonic saline (0.9% NaCl), followed by 400 mL of 4% paraformaldehyde (PFA) in 0.1 M phosphate buffer (PB, pH 7.4). Brains were then extracted and post-fixed in the same fixative solution as that used during perfusion for 24h at 4°C. Subsequently, brains were rinsed and cryoprotected by immersion in increasing concentrations of D-sucrose (10%, 20%, and 30% in 0.1 M PB) for 24 hours each at 4°C. Finally, brains were frozen using dry ice (solid carbon dioxide) and coronally sectioned at 40 μm with a cryostat (Leica, Wetzlar) for immunohistochemistry. According to the stereotactic atlas of the rat brain [22], sections encompassing the entire rostrocaudal extent of the VN (−9.84 mm to −13.08 mm from Bregma) were collected. Consecutive sections were systematically distributed into a 12-well plate containing cryoprotectant solution, generating 10–11 complete series per well and ensuring evenly spaced sampling across the full length of the VN.

### Immunohistochemistry

Immunohistochemical co-labeling was conducted based on protocols that have been previously validated [11]. Free floating sections were rinsed in 0.1M phosphate buffer saline (PBS, 3x5min) in multi-well plates and incubated for 1 h at room temperature (RT) in 5% bovine serum albumin/0.3% Triton X-100 for blocking and permeabilization. Primary antibody incubation was performed overnight at 4 °C with rabbit anti-IBA1 (1:2000, Fujifilcdi Wako, 019–19741), anti-GFAP (1:200, Dako, Z0334), or anti-Olig2 (1:500, Milipore, AB9610). Fluorescent secondary antibody, donkey anti-rabbit Alexa Fluor 488 (1:500, Invitrogen, A21206) was applied for 2 h at RT. DAPI staining (1:5000, Invitrogen, D1306) was performed before a light post fixation with 4% PFA. Floating brain sections were then washed with PBS and a second blocking was done by incubation (2 h, RT) in 10% SVF and DMEM. Then, heat-induced antigen retrieval in 2M HCl in PBS 0.5% Triton X-100 (30 min, 37°C), followed by neutralization with sodium tetraborate (pH = 8.5, 3 x 5 min, RT) was performed. After pH verification and PBS washes (3 x 5 min, RT), sections were incubated overnight at 4 °C with primary antibody mouse anti-BrdU (1:100, Dako, M0744 or 1:200, Proteintech, 66241-Ig). Alexa fluor 594 nm donkey anti-mouse secondary antibody was then used (1:500, Invitrogen, A21203) for 2 h at RT. Finally, brain sections were mounted onto SuperFrost/Plus glass slides (Epredia, J1800AMNZ) and air-dried before being mounted with Roti®Mount FluorCare antifade reagent (Carl Roth, HP19.1).

### Fluorescence image acquisition in the deafferented medial vestibular nucleus

Fluorescence images were acquired using a Zeiss LSM 710 NLO laser scanning confocal microscope equipped with a 63x/1.32NA oil immersion lens that delineates the region of interest by a 425.10μm² square. Multipanel images were acquired and stitched to encompass the full deafferented medial vestibular nucleus (MVN). Each multipanel consisted of orthogonal maximum intensity projections reconstructed from 2 to 7 optical slices (z-stacks), enabling spatial counting analysis of labeling across the tissue thickness. Confocal images and analysis revealed accumulation of the red BrdU signal within the green-labeled markers (for glial cells). In our study, BrdU labeling (red) marks nuclei of proliferating cells, whereas GFAP, IBA1, and Olig2 labeling (green) marks either the cytoplasm (GFAP, IBA1) or nuclei (Olig2). Therefore, from a technical standpoint, true colocalization can only be asserted for BrdU/Olig2 double labeling, where both signals overlap within the nucleus. In contrast, BrdU/GFAP and BrdU/IBA1 represent nuclear signal within cytoplasmic labeling. Z-stack images reconstructions were analyzed using the ZEN 2012 Black edition software (Carl Zeiss Microscopy GmbH, Germany). Image galleries displaying sequential focal planes across the 40µm tissue depth were generated (Fig 3A). Orthogonal views were also generated, showing cross-sectional slices along the x- and y-axes relative to the z-axis (Fig 3B). Finally, the z-stacks were reconstructed into 3D volumes and rotated in all planes to verify double labeling (Fig 3C and S2 Video, S3 Video).

**Fig 1:**
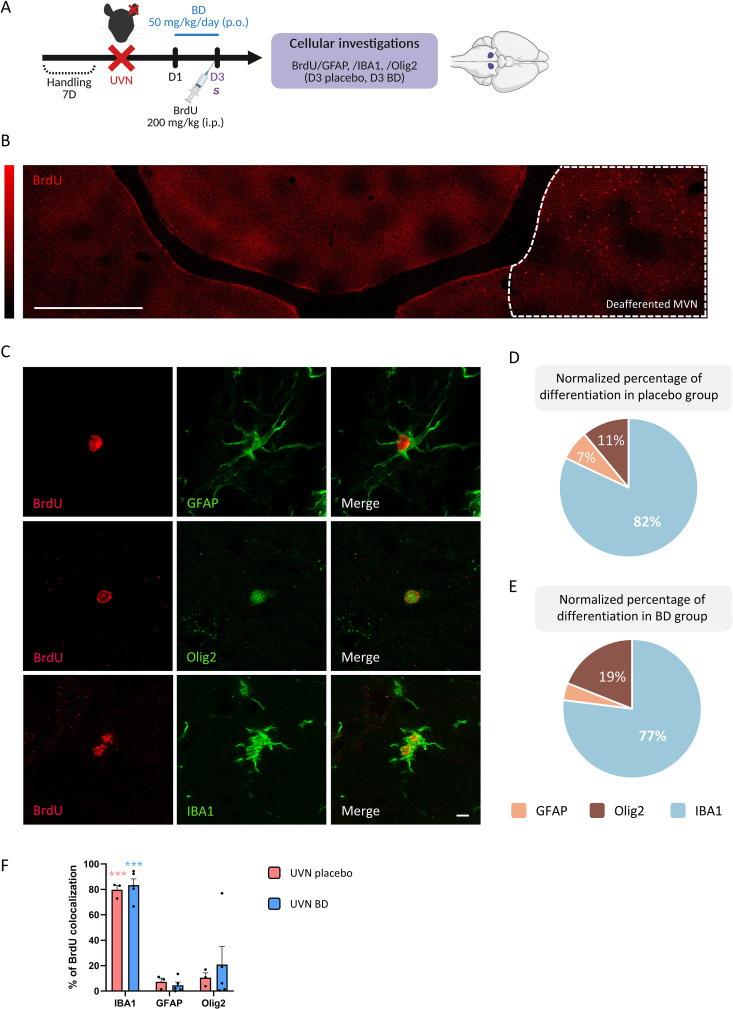
Study design, acute cell proliferation and differentiation in the deafferented medial vestibular nucleus following unilateral vestibular neurectomy. **A.** Study design used to assess the acute cell proliferation and differentiation following unilateral vestibular neurectomy (UVN). Betahistine dihydrochloride (BD) was given for 3 days (50 mg/kg/day, p.o) while UVN placebo group underwent gavage with an equivalent volume of 0.9% sodium chloride solution. UVN placebo (n = 3) and UVN BD (n = 5) received an injection of BrdU (bromodeoxyuridine, 200 mg/kg, i.p) on D3 and was killed 2 h after the injection to study cell proliferation and differentiation in the deafferented medial nucleus (MVN) by BrdU/GFAP,/IBA1,/Olig2 co-labelling. **B.** Multipanel views of orthogonal maximum intensity projections (4 optical sections) showing confocal immunostaining of BrdU+ cells (red) only in the deafferented (left) MVN 3 days after UVN and BD treatment; a similar pattern was observed in the UVN placebo group. A color scale indicates BrdU signal intensity ranging from 0 (black) to 4095 (red, maximal intensity), highlighting BrdU+ proliferating cells. Scale bar = 500 µm. The ipsilesional MVN is outlined with white dashed lines. **C.** Orthogonal maximum intensity projections of confocal images illustrating BrdU+ cells (red) co labeled with cell-specific markers (green) in the deafferented MVN: astrocytes (GFAP, 7 optical sections), oligodendrocytes (Olig2, 19 optical sections) and microglia (IBA1, 3 optical sections). Scale bar = 10 µm. D and **E.** Pie charts illustrating the percentage of GFAP+ , Olig2+ and IBA1+ cells among the BrdU+ cells in the UVN placebo **(D)** and BD **(E)** groups. These pie charts are based on the same data presented in the histograms but normalized to 100%. In the UVN BD group **(E)**, BrdU+/GFAP+ cells represent 4% of the total BrdU+ population. **F.** Quantitative analysis of the glial cell phenotype distribution in the UVN placebo and UVN BD groups. The histogram represents group means + SEM, and each point corresponds to the mean number of cells per animal analyzed (UVN placebo n = 3, UVN BD n = 5). Significant differences are indicated by * in red for comparison within the UVN placebo group (BrdU+/IBA1+ vs. BrdU+/GFAP+ and vs. BrdU+/Olig2+) and by * in blue for comparison within the UVN BD group. No significant difference was found between the UVN placebo and UVN BD groups. One-way ANOVA with Tukey’s post hoc test was used for glial phenotype distribution within each group and unpaired t-test was used for comparison between groups. ***p < 0.001.

**Fig 2:**
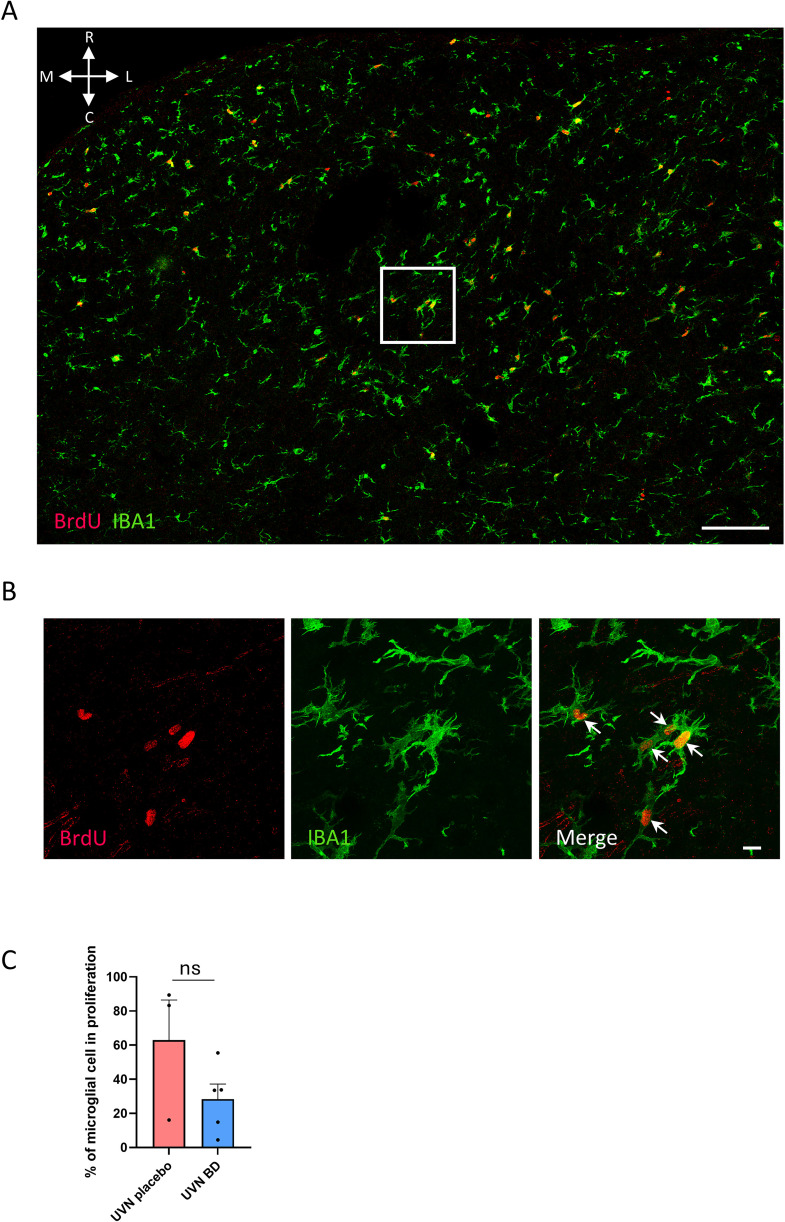
Acute and abundant microgliogenesis in the deafferented medial vestibular nucleus following unilateral vestibular neurectomy. **A.** Multipanel views of orthogonal maximum intensity projections (3 optical sections) showing confocal immunostaining of BrdU+ cells (red) and IBA1+ cells (green) in the deafferented (left) MVN D3 after UVN, with anatomical orientation indicated (R = rostral, C = caudal, M = medial, L = lateral). Scale bar = 100 µm. **B.** ROI of confocal image presented in A with orthogonal maximum intensity projections (62 optical sections) of BrdU+ cells (red) co-labeled with IBA1+ cells (green). White arrows show multiple microglia cells with incorporated BrdU. Scale bar = 10 µm. **C.** Quantitative analysis of proliferating microglia among the total microglial population in the MVN was performed in the UVN placebo and UVN BD groups. The histogram represents group means + SEM, and each point corresponds to the mean number of cells per animal analyzed (UVN placebo n = 3, UVN BD n = 5). No significant difference was found between the UVN placebo and UVN BD groups. Unpaired t-test was used for comparison between groups.

**Fig 3:**
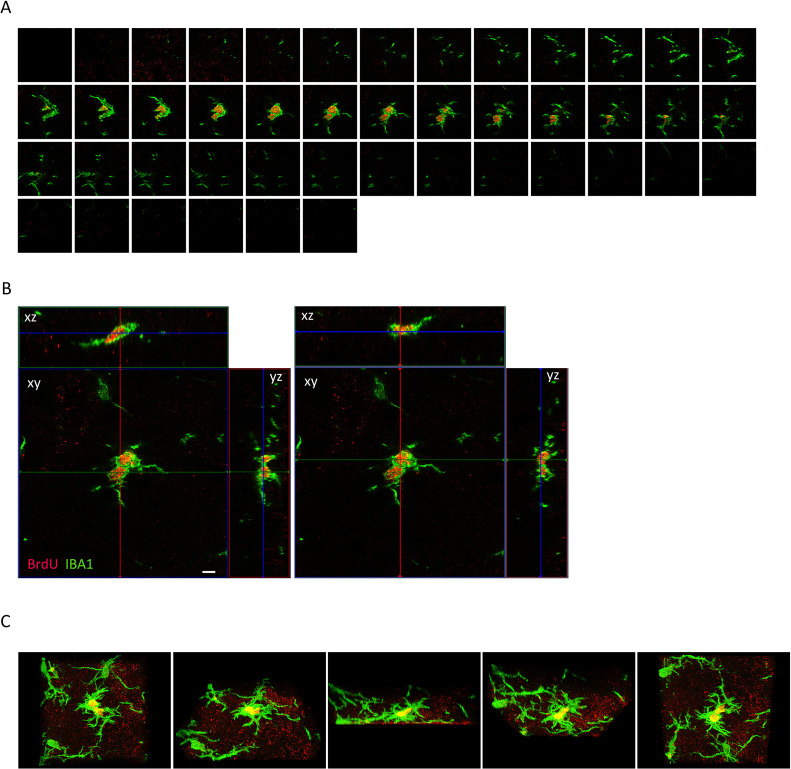
High resolution confocal images confirming acute microgliogenesis in our model. **A.** Sequential z-plane confocal sections (45 optical sections) of one proliferating cell with BrdU+ (red) and IBA1+ (green) immunostaining in the deafferented MVN at D3 following UVN. **B.** To confirm true co-labeling, orthogonal views (xy, xz and yz) of a single optical section shown in panel A were generated, displaying BrdU+ (red) and IBA1+ (green) immunostaining. Scale bar = 5 µm. **C.** Rotating sequential 3D views of this cell (45 optical sections) acquired using ZEN’s 3D display function.

### Cells count

The entire multipanel z-stack of the deafferented MVN was examined. Only sections of the MVN on the lesion (left) side were evaluated. Quantification of BrdU+/IBA1+, BrdU+/GFAP+ and BrdU+/Olig2+ cells was performed using a confocal imaging with a Zeiss LSM 710 NLO laser scanning microscope. Each multipanel image acquired as a z-stack composed of 2–7 optical slices (see Fluorescence image acquisition in the deafferented medial vestibular nucleus section) were used for counting. For each marker, immunoreactive positive cells in the MVN were counted using an integrated microscopic counting chamber that delineated the region of interest by a square of 425.10 μm^2^. Average numbers of cells per section, determined through ImageJ quantification, were used for analysis, according to previously validated protocols [12,23]. Image acquisition and cell counting were performed blindly by two independent investigators.

### Statistical analysis

Statistical analyses were performed using GraphPad Prism software (version 10, GraphPad Software). Data are presented as mean + SEM. For the analysis of glial phenotypes within each group (UVN placebo or UVN BD), a one-way ANOVA followed by Tukey’s post hoc test were used. Comparisons between groups (UVN placebo vs. UVN BD) for acute microgliogenesis and for the proportion of proliferating microglia cells among the total number of microglial cells were performed using unpaired t-tests. p values of < 0.05 were considered statistically significant.

## Results: Acute microgliogenesis is restricted to the deafferented vestibular nuclei

To study acute cell proliferation lineage, a BrdU injection at D3 post-UVN was performed followed by immunostaining (see study design, Fig 1A). In this pilot study, BrdU immunostaining in both MVN is shown for an animal from the UVN BD group in Fig 1B; a similar pattern was observed in the UVN placebo group. Cell proliferation was largely confined to the deafferented MVN for UVN D3 placebo or UVN D3 BD groups, whereas only sparse BrdU+ cells were occasionally detected in adjacent nuclei or in the contralateral MVN. To study acute glial differentiation, we used BrdU marker co-labeled with astrocytes (GFAP), oligodendrocytes (Olig2) and microglia (IBA1) markers. These immunostainings are presented in Fig 1C. For both groups, the number of BrdU+/IBA1+ cells is favored (*** p < 0.001 for both groups) at the expense of BrdU+/GFAP+ or BrdU+/Olig2+ cells (Fig 1D and 1E, 1F). No statistical differences were found in BrdU+/IBA1+ cells between our two groups (Fig 1F) and numerous BrdU+/IBA1+ cells were detected across the entire parenchyma of the deafferented MVN (Fig 2A, 2B). This suggests a predominant microglial origin of newborn cells within the deafferented MVN at 3 days post-UVN, whereas astrocytic (BrdU+/GFAP+) and oligodendrocytic (BrdU+/Olig2+) newborn cells represent only a small minority (from 4 to 19%). Among the total number of microglial cells present in the MVN, approximately 63% and 28% were proliferating in the UVN placebo and UVN BD groups, respectively, with no significant difference between groups (Fig 2C). To further investigate this unique phenomenon, we analyzed the microglial cell with BrdU+ /IBA1+ staining presented in Fig 1C. This cell was analyzed in 45 optical sections collected in 0.46 µm intervals (Fig 3A and S1 Video). As expected, BrdU staining is present in the nucleus of the microglial cell (IBA1 staining) as shown in orthogonal views between planes (xy, xz, and yz; see Fig 3B) and in the 3D image and video reconstruction (Fig 3C, S2 Video, S3 Video). Several other BrdU+/IBA1+ staining are presented in the Supplementary figures (see S1 Data). Notably, we repeatedly observed closely apposed BrdU+ nuclei within IBA1+ microglial cells, suggesting local proliferation events. Such features were not observed in BrdU+/GFAP+ or BrdU+/Olig2+ co-labeling cells. This acute microgliogenesis have been observed in all deafferented VN and is not restricted to MVN (S2 Data). Considering these preliminary results, acute and abundant microgliogenesis may represent an intrinsic adaptative plasticity of the VN following UVN.

## Discussion

In the UVN model, the cell proliferation peak occurs at day 3 post-lesion [17] and is not observed in sham-operated control groups [11,13]. A few BrdU+ cells have been described in control animals only under experimental conditions involving repeated BrdU administrations or extended survival times after BrdU injection [24–26]. This cell proliferation has been consistently reported to occur exclusively in the deafferented VN [10,11,13,17]. In parallel, glial activation is reported in the same region [7,8,13,15,27]. Previous studies have demonstrated neuroglial differentiation (GABAergic neurons, astrocytes, microglia and oligodendrocytes) and cell survival at later stages, up to one month post-lesion [9–11,13]. However, proliferating glial cells during the acute phase of vestibular syndrome has never been studied in models of vestibular pathology. These early changes may be critical for the onset of vestibular compensation, as this acute phase is decisive for the overall recovery process. Our study aims to provide preliminary insights into the proliferative and differentiation processes occurring at D3 post-UVN in the deafferented VN.

Although BD favored microglial differentiation and improved vestibular compensation at later stages (D30 post-lesion) compared to the untreated lesioned group [13], no difference were observed in the glial cell phenotype between the BD and untreated groups at D3 post-lesion. In addition, both groups exhibited robust acute cell proliferation in the deafferented MVN [13], with a moderate yet significant increase in the BD-treated group compared to placebo. This aspect has already been discussed in our previous work [13]. These findings suggest that BD acts at later stages by modulating cell fate toward a microglial phenotype and sustaining acute microgliogenesis over time. As the long-term effects of BD have already been addressed and discussed in our previous study [13], this pilot study focuses specifically on the acute microgliogenesis elicited by the lesion itself.

Astrocytes and oligodendrocytes primarily derive from neural stem or precursor cells [28–30], and their differentiation process is slower than microglial proliferation, which arises from a pre-existing pool of resident cells [31,32]. Thus, new microglia are likely the first responders in the early post-lesion phase of the vestibular syndrome. While newly generated astrocytes and oligodendrocytes may also contribute at this early stage, their limited early proliferation might indicate a more substantial role during post-acute or later stages of the vestibular syndrome. The presence of BrdU+ /IBA1+ cells broadly distributed throughout the VN parenchyma and not limited to periventricular regions (IV^th^ ventricle), indicates microglial proliferation within the VN. This distribution pattern challenges the notion of microglial migration and proliferation from other brain regions. Moreover, given the short interval between BrdU injection and tissue collection (2 h), infiltration and differentiation of blood-derived monocytes seem unlikely [33]. Given that vestibular lesion induces an inflammatory environment [34], local microglial proliferation may involve the colony stimulating factor-1 receptor (CSF-1R), which can be triggered by IL-34, a pro-inflammatory cytokine [35–40]. Other pro-inflammatory cytokines, including TNF-α, are also known to promote cell proliferation [41]. Importantly, the phenotype of newly generated microglia—whether pro- or anti-inflammatory— remains undetermined, and could differ between our treated and untreated conditions, as histamine’s effects are known to depend on the local inflammatory environment [see for review 42]. Supporting the hypothesis of endogenous microgliogenesis, it should be emphasized that microglia can self-renew and repopulate from a residual pool of resident cells following depletion [43,44].

Endogenous microgliogenesis, primarily resulting from the proliferation of resident microglial cells, has been described in several pathological conditions beyond the vestibular lesion model. Acute brain injuries such as stroke, optic nerve lesion, or perforant path transection induce rapid microglial activation and proliferation within hours to days after injury [45–49]. Similarly, viral-induced epileptic models such as Theiler murine encephalomyelitis virus (TMEV) also display pronounced microgliogenesis [50]. Consistent with these findings, in an axonal lesion model, lesion-reactive microglia represented most of proliferating cells 1h after BrdU injection, with 25% of all microglial cells proliferating 3 days post-lesion [45]. In our UVN model, we observed comparable microglial proliferation. These studies collectively indicate that microglial proliferation in response to injury mainly arises from the *in situ* proliferation of resident microglia, underscoring a conserved mechanism of endogenous microgliogenesis across various pathological conditions. Within this context, our work provides a first pilot description of acute microgliogenesis following UVN, which opens new perspectives on the nature and significance of this early cellular response in vestibular compensation, which will be discussed below.

Can these newborn microglia be functional? As highlighted by Zhan et al., newly generated microglia are morphologically mature and might be potentially functional [51] and thus participate for vestibular compensation in our model.

Could microglia contribute to the onset of vestibular compensation or rather to the expression of the vestibular syndrome? If microglia are involved in vestibular compensation, their capacity to modulate neuronal activity through direct contact with dendritic spines, thereby enhancing synaptic transmission and synchronizing neuronal networks [52] would be of primary importance. Microglia would participate in the restoration of homeostatic excitability within the deafferented VN that is the first key objective to promote functional recovery. Moreover, microglia express glutamate, GABA, purinergic, and cholinergic receptors [53–57], all relevant to vestibular signaling [58], illustrating microglia’s capacity to respond to neurotransmitters and adapt to environmental changes. Furthermore, receptor expression is context-dependent [55], highlighting the plasticity of microglia in response to environmental changes—a property that could be particularly relevant after UVN. Yet, microglia could either support recovery or, if overactivated, impair the endogenous plasticity [59] required for compensation. The phenotype of newly generated microglia could influence their contribution to vestibular compensation, as overactivation of pro-inflammatory states might hinder cellular plasticity, whereas anti-inflammatory or neuroprotective states could facilitate functional recovery.

Finally, could microglia play similar roles during the acute and compensated phase of the vestibular syndrome? The acute phase of the vestibular syndrome occurs within the first week after UVN and is marked by the expression of sequential plasticity mechanisms in the deafferented VN that support functional recovery [18]. Given their own plasticity, microglia in the acute phase might support compensation through BDNF release, a key factor accelerating vestibular compensation [20]. This aligns with the marked ipsilesional BDNF increase following vestibular lesion [60] and the BDNF-mediated downregulation of KCC2 within the VN [13,23]. KCC2 downregulation modulates neuronal excitability by altering GABAergic signaling, therby increasing the excitability of neurons [61]. Since KCC2 downregulation is transient during the acute phase [12,13,23], microglia at the compensated stage may cease BDNF release and shift toward their next sequential function to sustain vestibular compensation in the UVN placebo group. Given their inherent plasticity, microglia may adopt different phenotypes at the acute and compensated stages, potentially contributing to stage-specific mechanisms of recovery. In the longer term, microglia may dedifferentiate into excitatory neurons [62] that can play a critical role in sustaining long-term excitability within the deafferented VN.

## Conclusion

This pilot study provides preliminary evidence that microglia display remarkable plasticity within the deafferented VN following UVN. These findings raise the question of whether acute microgliogenesis may be functional and exert a neuroprotective role during the acute phase of the vestibular syndrome. Further investigations combining cytokine profiling, pharmacological modulation of microglia (e.g., minocycline to inhibit or pexidartinib to deplete microglia), and behavioral assessments will be essential to clarify its potential functional significance in this model. Given the small sample size, these observations should be considered preliminary, and replication by independent research teams will be critical to validate and expand upon these initial findings.

## Supporting information

S1 Video Sequential visualization of a complete confocal z-stack (45 optical sections) of BrdU+ (red) and IBA1+ (green) immunostaining in the deafferented medial vestibular nucleus (MVN) at 3 days post-lesion. The z-stack was acquired with a confocal microscope and is displayed at 6 frames per second to visualize the spatial distribution of proliferating microglia through the depth of the tissue.
(AVI)

S2 Video 3D rotation of a confocal z-stack (45 optical sections, 3 frames/s) showing BrdU+ (red) and IBA1+ (green) immunostaining in the deafferented medial vestibular nucleus (MVN) at 3 days post-lesion. The 3D rendering was generated using the ZEN software “3D display” tool. The video presents successive angular views to visualize the spatial distribution of BrdU+ nuclei within IBA1+ microglia rotating around the y-axis across the reconstructed volume.
(AVI)

S3 Video 3D rotation of a confocal z-stack (45 optical sections, 3 frames/s) showing BrdU+ (red) and IBA1+ (green) immunostaining in the deafferented medial vestibular nucleus (MVN) at 3 days post-lesion. The 3D rendering was generated using the ZEN software “3D display” tool. The video presents successive angular views to visualize the spatial distribution of BrdU+ nuclei within IBA1+ microglia rotating around the x-axis across the reconstructed volume.
(AVI)

S1 Data High resolution z-stack images confirming acute microgliogenesis in our model. **A.** Single optical section of one proliferated microglial cell showing BrdU+ nucleus (red) within IBA1+ microglial cell (green) in the deafferented MVN, 3 days after lesion. Scale bar = 5 µm. **B.** Sequential z-plane confocal sections (38 optical sections) of this cell illustrating BrdU+ (red) and IBA1+ (green) immunostaining. **C.** To confirm true co-labeling, orthogonal views (xy, xz and yz) of a single optical section shown in panel A were generated, displaying BrdU+ (red) and IBA1+ (green) immunostaining. **D.** Rotating sequential 3D views of this cell (38 optical sections) acquired using ZEN’s 3D display function.
(TIF)

S2 Data High-resolution z-stack multipanel images showing acute microgliogenesis across all other vestibular nuclei following unilateral vestibular neurectomy. **A-C.** Multipanel views of orthogonal maximum intensity projections (4 optical sections) showing confocal immunostaining of BrdU+ cells (red) and IBA1+ cells (green) in the deafferented (left) vestibular nuclei (VN) 3 days after unilateral vestibular neurectomy (UVN). Confocal images from the BD group are presented but a similar pattern was observed in the UVN placebo group. For each panel, the corresponding Paxinos atlas section is shown on the left, with the ROI indicated by a red square at three different bregma levels: −10.08 mm (**A**), −11.04 mm (**B**) and −11.76 mm (**C**) for the superior (SVN), lateral (LVN) and inferior (IVN) vestibular nuclei, respectively. On the right, the corresponding multipanel z-stack images show the deafferented VN outlined with white dashed lines. Anatomical orientation indicated (R = rostral, C = caudal, M = medial, L = lateral). 4V = 4^th^ ventricle. Scale bar = 200 µm.
(TIF)

## Abbreviations

BD: Betahistine dihydrochloride
BrdU: bromodeoxyuridine
MVN: medial vestibular nuclei
UVN: unilateral vestibular neurectomy
VN: vestibular nuclei
