# Supplementary figures and images for: A pilot study on early microgliogenesis following unilateral vestibular neurectomy: A key player in vestibular compensation?
Source: PLoS One. 2026 Jan 7;21(1):e0339767. doi: 10.1371/journal.pone.0339767 (PMC12779071; doi:10.1371/journal.pone.0339767)

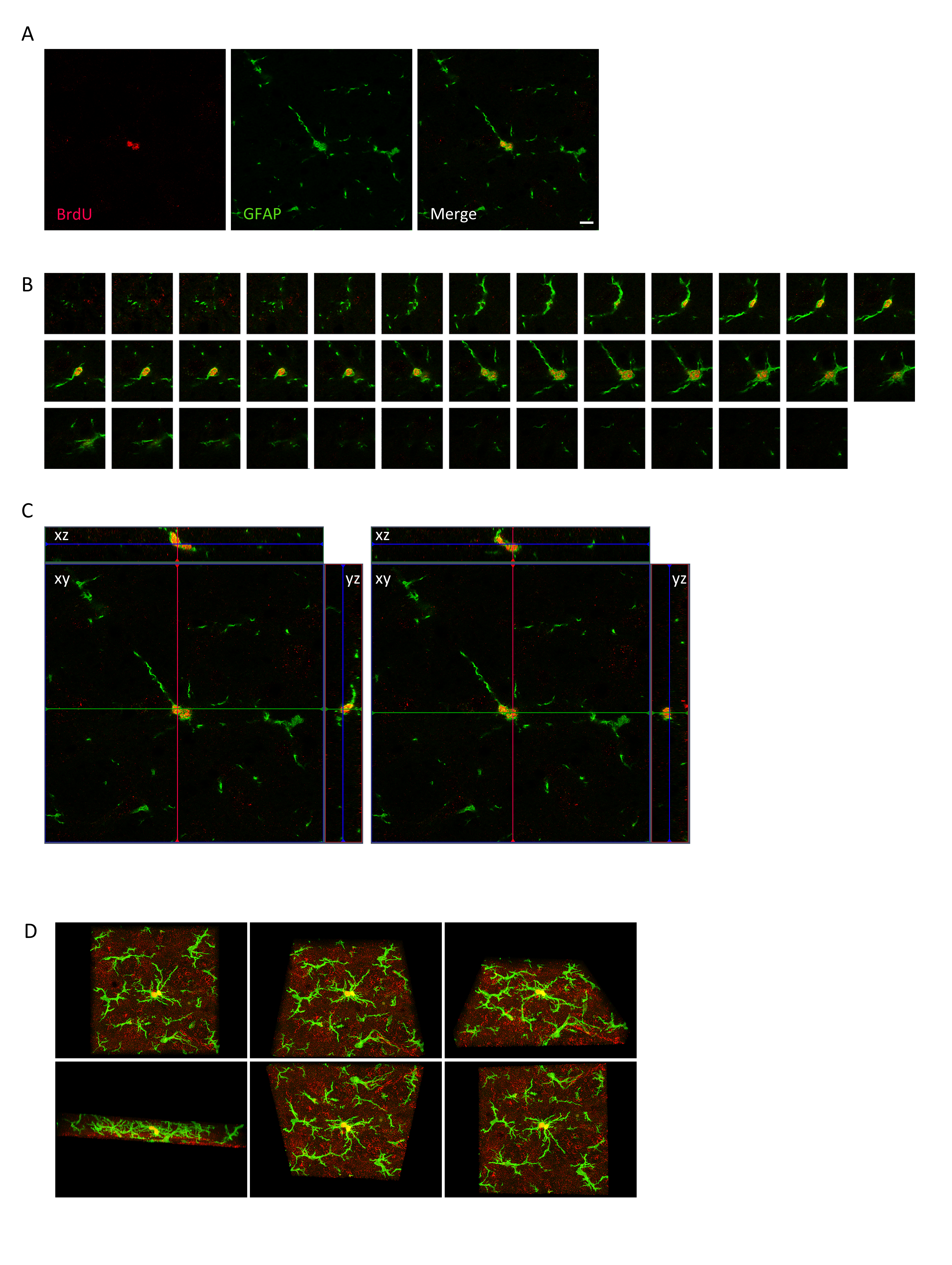

Supplement: S1 Data — A. Single optical section of one proliferated microglial cell showing BrdU+ nucleus (red) within IBA1+ microglial cell (green) in the deafferented MVN, 3 days after lesion. Scale bar = 5 µm. B. Sequential z-plane confocal sections (38 optical sections) of this cell illustrating BrdU+ (red) and IBA1+ (green) immunostaining. C. To confirm true co-labeling, orthogonal views (xy, xz and yz) of a single optical section shown in panel A were generated, displaying BrdU+ (red) and IBA1+ (green) immunostaining. D. Rotating sequential 3D views of this cell (38 optical sections) acquired using ZEN’s 3D display function. (TIF) [file pone.0339767.s004.tif]

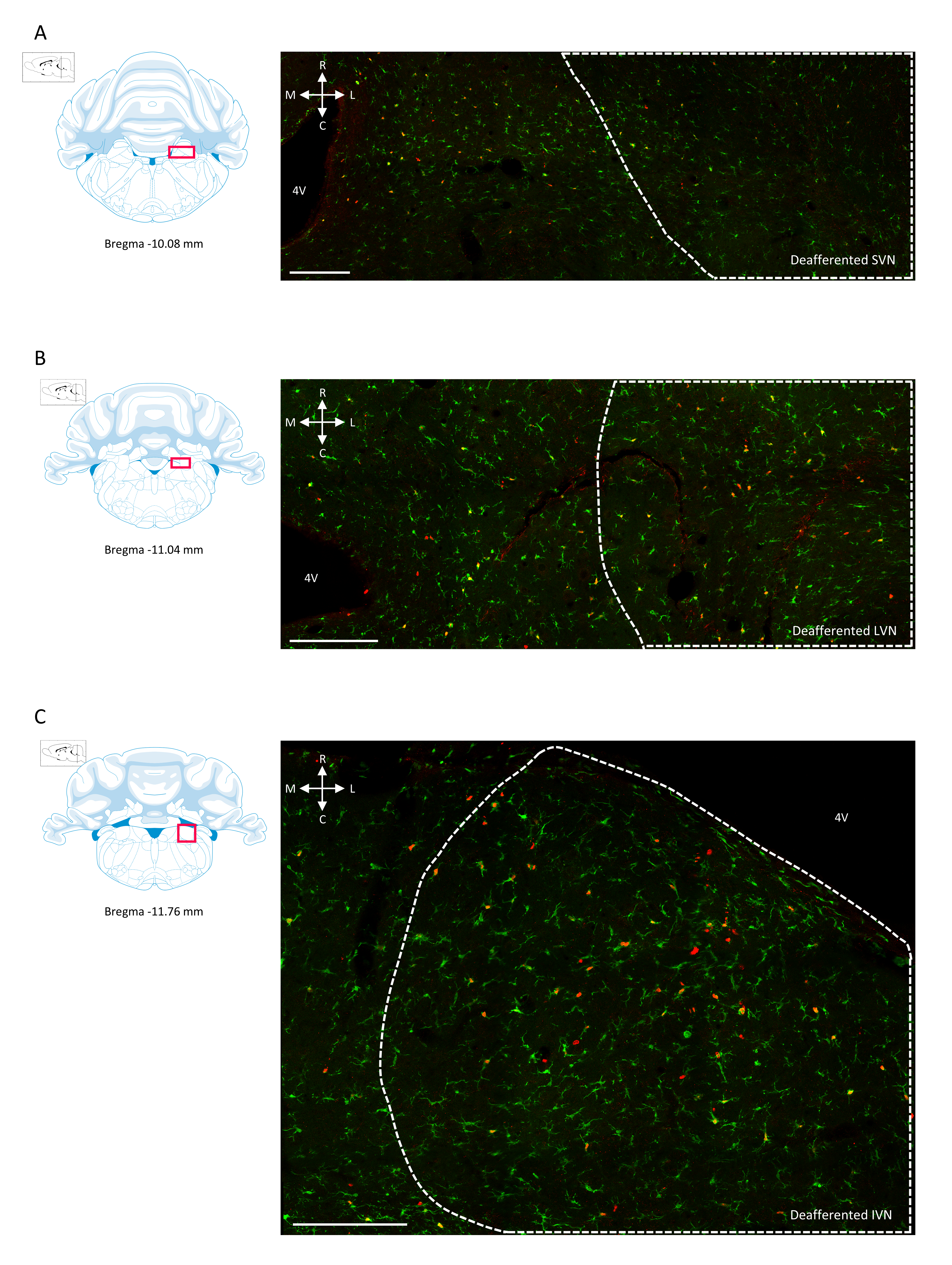

Supplement: S2 Data — A-C. Multipanel views of orthogonal maximum intensity projections (4 optical sections) showing confocal immunostaining of BrdU+ cells (red) and IBA1+ cells (green) in the deafferented (left) vestibular nuclei (VN) 3 days after unilateral vestibular neurectomy (UVN). Confocal images from the BD group are presented but a similar pattern was observed in the UVN placebo group. For each panel, the corresponding Paxinos atlas section is shown on the left, with the ROI indicated by a red square at three different bregma levels: −10.08 mm (A), −11.04 mm (B) and −11.76 mm (C) for the superior (SVN), lateral (LVN) and inferior (IVN) vestibular nuclei, respectively. On the right, the corresponding multipanel z-stack images show the deafferented VN outlined with white dashed lines. Anatomical orientation indicated (R = rostral, C = caudal, M = medial, L = lateral). 4V = 4th ventricle. Scale bar = 200 µm. (TIF) [file pone.0339767.s005.tif]
